# Silicon Carbide Photonic Crystal Photoelectrode

**DOI:** 10.1002/advs.202415552

**Published:** 2025-03-17

**Authors:** Xiwen Zhang, Sajeev John

**Affiliations:** ^1^ Department of Physics University of Toronto 60 Saint George Street Toronto Ontario M5S 1A7 Canada

**Keywords:** photoelectrochemical cell, photoelectrode, photonic crystal, silicon carbide, water splitting

## Abstract

The immense challenge of large‐scale implementation of photoelectrochemical (PEC) water splitting and carbon fixation lies in the need for a cheap, durable, and efficacious photocatalyst. Cubic silicon carbide (3C‐SiC) holds compelling potential due to its auspicious band positions and high‐volume, high‐quality, single crystal industrial manufacturing, but is hindered by its inferior light absorptivity and anodic instability. A slanted parabolic pore photonic crystal (spbPore PC) architecture with graphitic carbon nitride (g‐CN), nickel(II) oxide (NiO), or 6H silicon carbide protective coatings is proposed to overcome the drawbacks of 3C‐SiC photoelectrodes. A 30 µm‐ and 62 µm‐thick 3C‐SiC spbPore PC of lattice constant 0.8 µm demonstrates maximum achievable photocurrent density (MAPD) of 9.95 and 11.53 mA cm^−2^ in the [280.5, 600] nm region, respectively, representing 75.7% and 87.7% of the total available solar photocurrent density in this spectral range. A 50 nm‐thick g‐CN or NiO coating forms type‐II heterojunctions with the 3C‐SiC spbPore PC, facilitating the charge transport and enhancing the corrosion resistivity, all together demonstrating the MAPD of 9.81 and 10.06 mA cm^−2^, respectively, for 30 µm‐thick PC. The scheme advances the low‐cost, sustainable, real‐world deployment of PEC cells for green solar fuel production.

## Graphical abstract

A 3C‐SiC photoelectrode with a slanted parabolic pore photonic crystal architecture enhances photogeneration for efficient photoelectrochemical water splitting through the remarkable light‐trapping capabilities of photonic crystal slow‐light modes. Protective coatings of g‐CN, NiO, or 6H‐SiC further boost corrosion resistance and charge transport, providing a pathway for economic, large‐scale green solar fuel production.

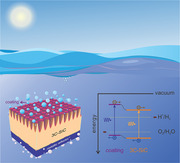

## 1 Introduction

Within the past seven decades, humans have consumed 1.5‐times the energy of the entire preceding 11.7 thousand years, largely through the combustion of fossil fuels. Such explosive expenditure of carbon‐based energy has become an artificial geological force, profoundly altering Nature at the planetary scale and arguably opening a new epoch‐level interval, Anthropocene, in the earth's history. During this period, the atmospheric CO_2_ concentration experienced a sharp increase to the present level that surpasses any time in the past 3 million years.^[^ ^1^, ^2^, ^3^ ^]^ Today, the consequent long‐lasting volatile climate change plausibly becomes “the biggest threat modern humans have ever faced” that may result in a security collapse of the habitable environment including food and fresh water.^[^ ^4^ ^]^ This immense challenge to contemporary civilization inexorably calls for a reform of fuels from carbon‐rich to carbonless materials such as hydrogen, and economical ways to recycle the already released CO_2_ in the atmosphere. Photoelectrochemical (PEC) cells hold promise for sustainable solar fuel generation and carbon fixation by splitting water into oxygen and hydrogen gases^[^ ^5^ ^]^ and converting CO_2_ to more reduced chemical species,^[^ ^6^, ^7^ ^]^ respectively. However, they are still far from large‐scale deployment compared with the already commercialized silicon photovoltaics.

In addition to the common requisites on solar energy harvesting devices of i) cost‐effectiveness and abundance, ii) environmental safety, iii) high stability, iv) high solar photon absorptivity, and v) small charge carrier losses, the photoelectrode semiconductor in PEC cell has to meet extra conditions including vi) high photoreactivity, vii) small bandgaps and straddling bands positions with respect to the redox potentials, and stability in aqueous environment (corrosion resistivity). Unfortunately, so far there has been no material satisfying all these requirements. For example, the widely used TiO_2_ photoelectrode suffers from wide bandgap and weak absorptivity. These stringent requisites impose great challenges to the real‐world implementation of PEC solar fuel photoproduction and carbon dioxide photoreduction. Consequently, achieving economic and environmental feasibility ultimately demands a compromise among these requirements. The physical and chemical properties of silicon carbide, combined with its abundance and industrial fabrication capabilities, present an alternative to traditional approaches that often emphasize efficiency at the expense of other factors. In contrast, the approach suggested in this work builds on cost‐effectiveness and environmental safety from the outset, and compensates for the inevitable trade‐offs in photogeneration and stability (in the case of photoanode) through the careful design of the PEC electrode morphology.

Indeed, the success of silicon electronics and photonics exemplifies the essential importance of material security, especially abundance, of the underlying element and the industrializability of the working semiconductor. After all, solar fuel production and carbon capture are not only scientific subjects but also equally economic issues, and conditions like i) and ii) are very much inexpugnable. Si is the second‐most abundant element in the earth's crust with an annual production of nine million tonnes in 2023.^[^ ^8^ ^]^ While Si absorbs light strongly in the visible spectrum, it has an insufficient bandgap to split water and undergoes photodegradation in an aqueous solution. We require low‐cost semiconductors amenable to high‐quality, high‐volume, industrial manufacturing that may play a similar role of Si in photovoltaics, in PEC cells.

Silicon carbide (SiC) is a well‐studied and cost‐efficient semiconductor for high‐power, high‐speed, and high‐temperature electronic devices due to its excellent electronic, thermal, and mechanical properties.^[^ ^9^, ^10^ ^]^ Both silicon and carbon are abundant on earth, and high‐quality SiC single crystals are commercially available. While its PEC applications have been investigated in the early years of solar fuel photoproduction,^[^ ^11^, ^12^ ^]^ it is in the past decade that the cubic polytype of silicon carbide (3C‐SiC) gained much research interest for water splitting and CO_2_ photoreduction.^[^ ^13^, ^14^, ^15^, ^16^, ^17^, ^18^, ^19^, ^20^, ^21^, ^22^, ^23^, ^24^, ^25^, ^26^ ^]^ Generally speaking, 3C‐SiC satisfies most requirements (i–vii), except for two major weaknesses. First, despite the small bandgap ∼2.35 eV, 3C‐SiC has very low photon absorptivity in the UV to the visible spectral range due to the indirect nature of this bandgap. Although material modifications by excess doping generally improve absorptivity, this may degrade the overall light‐harvesting efficiency by promoting charge carrier recombination. Second, silicon carbide undergoes anodic oxidation for oxygen evolution, degrading its photocatalytic reactivity and long‐term durability by forming surface silicon dioxide, carbonates, carbon dioxide, etc.^[^ ^13^, ^17^, ^27^ ^]^

The purpose of this work is to propose a 3C‐SiC photonic crystal (PC) photoelectrode to fulfill the complete wishlist (i–vii) for high‐efficiency PEC cell. The weak absorptivity of 3C‐SiC, caused by its indirect bandgap, is well‐suited for PC light‐trapping enhancement.^[^ ^28^, ^29^, ^30^, ^31^, ^32^, ^33^, ^34^ ^]^ In particular, we consider a slanted parabolic‐pore (spbPore) structure,^[^ ^35^, ^36^ ^]^ coated by thin layers of graphitic carbon nitride (g‐CN), nickel(II) oxide (NiO), or 6H polytype of silicon carbide (6H‐SiC), for resolving the drawbacks of 3C‐SiC. Photonic crystal light‐trapping significantly enhances the light absorptivity without resorting to material modification that compromises the charge transport, whereas the surface coatings reinforce the chemical stability (of the photoanode) to corrosion effects. In addition, the composite architectures form type‐II semiconductor heterojunctions facilitating the electron‐hole separation, and their curvy morphology enriches the electrode‐electrolyte interface area for surface redox reactions.

Experimentally, inverse opal PCs have been implemented to improve the light absorption for photocatalysis,^[^ ^37^, ^38^ ^]^ and various other types of PCs have been proposed to enhance the photocatalytic and solar fuel production efficiency for lightly doped TiO_2_.^[^ ^32^, ^33^, ^34^ ^]^ The strong light‐trapping effect in PCs^[^ ^39^ ^]^ enormously increases the light‐matter interaction time, enabling large light absorptivity even for weak absorption coefficient. By judiciously designing the PC structure, the photon absorption and carrier conduction considerations can be decoupled. Suitable photonic crystals can also branch electron and hole diffusions, resolving the mismatch of the minority carrier diffusion, majority carrier diffusion, and photon absorption lengths.^[^ ^33^, ^34^ ^]^ Recent advances in tunable and responsive PCs^[^ ^40^ ^]^ have enabled versatile control over the optical properties of these materials.^[^ ^41^, ^42^, ^43^ ^]^

We demonstrate that a 30 µm‐thick, lightly‐doped, 3C‐SiC spbPore PC with lattice constant 0.8 µm, parallel pore depth 1 µm, slant angle 1.75°, 50 nm‐thick SiO_2_ passivation layer, placed on a reflective bottom contact, can reach maximum achievable photocurrent density [MAPD, see Equation (1) for definition] of 9.95, 9.81, and 10.06 mA cm^−2^ with no coating, 50 nm‐thick g‐CN coating and 50 nm‐thick NiO coating, respectively, in the wavelength range of [280.5, 600] nm. As the total available MAPD in this spectral range is 13.146 mA cm^−2^, this represents ∼75% of the total available photocurrent density. Further increasing the thickness of the 3C‐SiC PC to 62 µm, close to the highest reported hole diffusion length, results in an MAPD of 11.53 mA cm^−2^ (with slant angle 0° and no coating).

## 2 Material and Structural Models

### 2.1 Optical and Electronic Properties of 3C‐SiC

**Figure 1:**
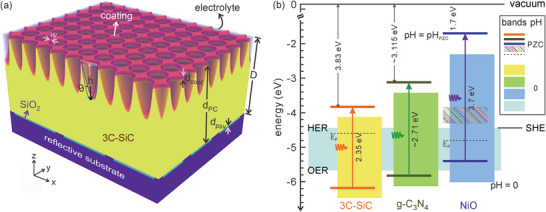
Illustration of silicon carbide photonic crystal photoelectrode. a) The photoelectrode is composed of 3C‐SiC slanted parabolic‐pore layer on bulk 3C‐SiC, with g‐CN, NiO, or 6H‐SiC surface coating and SiO_2_ rear passivation layer, deposited on a highly reflective conductive substrate and submerged in aqueous solution. Periodic metal protrusions separated by a distance close to the hole diffusion length of 3C‐SiC connect the bulk 3C‐SiC and the metal substrate for charge transport, while minimizing surface recombination of carriers. The slanted parabolic pore is obtained from a rotation of a normal pore of depth *h* around *y*‐axis pivoting at the surface edge by an angle θ. The PC consists of a square lattice of such (slanted) pores with periodicity *a*. The thicknesses of the surface coating, 3C‐SiC and SiO_2_ layers measured along vertical direction *z* are *d*_coat_, *d*_PC_, and *d*_psv_, respectively. The total thickness of the optically active materials is *D* = *d*_coat_ + *d*_PC_. b) Band positions of n‐type 3C‐SiC, p‐types g‐C_3_N_4_ and NiO in empty space or, equivalently, point of zero charge aqueous environments (thick horizontal lines), and those together with hydrogen evolution reaction (HER) and oxygen evolution reaction (OER) energies in pH = 0 electrolyte (colored bands). In other words, the separately color‐filled bands for each material depict the energies in the pH = 0 aqueous environment, not in physical vacuum. The pH_PZC_ is 4.9 for 3C‐SiC,^[^ ^52^ ^]^ 5.2 for g‐CN,^[^ ^53^ ^]^ and 10.3 for NiO.^[^ ^54^ ^]^ The physical vacuum (empty space), a potential level of 0 V, is defined as −4.44 V with respect to SHE. This latter energy coincides with the minimum energy of an electron in a pH = 0 solution to cause HER. The illustrated band positions assume flat band conditions, following *E* = *E*_PZC_ + 0.059(pH − pH_PZC_) eV, where *E*_PZC_ is the band positions of semiconductors in electrolytes of pH = pH_PZC_ (equated with the band positions in an empty space). The second term of *E* is due to the formation of a Helmholtz layer on the semiconductor‐electrolyte interface,^[^ ^48^, ^49^, ^55^, ^56^, ^57^ ^]^ causing a static electric field to occur in the surface region of the semiconductor. In general, energy shifting rates (due to this field) with respect to SHE other than 59 meV pH^−1^ are also possible.^[^ ^49^, ^56^ ^]^ In non‐flat band conditions, more extensive space charge regions near the semiconductor surfaces emerge and the concomitant band bendings (not shown in the figure) may or may not vary with pH,^[^ ^58^ ^]^ depending on surface reactions. *E*_*F*_ (dashed lines) denotes typical experimental Fermi levels of the semiconductors, and the rainbow‐shaded region of NiO corresponds to its in‐gap states of d‐d transitions, both illustrated in an empty space (or equivalent pH_PZC_) environment.

Silicon carbide is widely used as a substrate of light‐emitting diode and electronics operating in high‐power, high‐frequency, and high‐temperature conditions.^[^ ^9^, ^10^ ^]^ It exhibits a variety of polytypes characterized by different stacking orders of the Si‐C bilayers. Among these polytypes, 3C‐SiC has the lowest bandgap of ∼2.35 eV.^[^ ^44^, ^45^, ^46^ ^]^ Its valence band maximum (VBM), conduction band minimum (CBM) and Fermi level are located at ∼−6.18 eV, ∼−3.83 eV, and ∼−4.6 eV, respectively, relative to the vacuum level,^[^ ^45^, ^47^ ^]^ which is 4.44 eV above the potential of the standard hydrogen electrode (SHE). As described in **Figure** **1**, the SHE potential corresponds to the potential of the hydrogen evolution reaction (HER) at pH = 0. In aqueous solution, the band positions shift with pH due to the potential drop across the Helmholtz layer, which forms as a result of surface charge adsorption.^[^ ^48^, ^49^ ^]^ The point of zero charge (PZC, or explicitly pH_PZC_) value, a pH at which such potential drop across the Helmholtz layer is zero,^[^ ^50^ ^]^ ranges from a pH of 3 to 5.2^[^ ^51^ ^]^ for silicon carbide. The value of pH_PZC_ = 4.9 is adopted for 3C‐SiC^[^ ^52^ ^]^ to estimate the band positions shift, taken to be 59 meV pH^−1^, under pH_PZC_ and pH = 0 (see Figure 1b). These band potentials estimated in a general aqueous solution, with pH ≠ pH_PZC_, are not to be confused with those using physical vacuum (empty space) as the reference energy. The low bandgap together with the straddling band positions on the redox potentials of H^+^/H_2_ and O_2_/H_2_O makes 3C‐SiC ideally suited for PEC cell application.^[^ ^16^, ^18^ ^]^ The optical models of 3C‐SiC are detailed in Appendix B.1. Unless otherwise specified, Model‐L from Appendix B.1, representing a high‐quality crystal with low doping concentration, is used throughout this work.

3C‐SiC has excellent charge transport properties. The longest reported lifetime of photogenerated charge carriers is 8.2 µs at a doping concentration ∼10^15^ cm^−3^, with the corresponding bulk lifetime ∼10–15 µs.^[^ ^59^ ^]^ If the surface recombination is suppressed by proper surface passivation, the minority (hole) and majority (electron) carrier diffusion lengths demonstrate exceptional scales of *L*_*p*_ ∼ 55–67 µm and *L*_*n*_ ∼ 165–201 µm, respectively (assuming the hole diffusion coefficients *D*_*p*_ = 3 cm^2^ s^−1^^[^ ^59^ ^]^ and electron diffusion coefficient *D*_*n*_ = 9*D*_*p*_^[^ ^60^, ^61^ ^]^). Even considering a much smaller *D*_*p*_ = 1 cm^2^ s^−1^ and carrier lifetime 8.2 µs, the hole diffusion length still reaches *L*_*p*_ ∼ 29 µm (see Appendix A for more details). This is orders of magnitude higher than that of the widely used photocatalyst TiO_2_ which has *L*_*p*_ ∼ 10–300 nm^[^ ^62^, ^63^, ^64^, ^65^ ^]^ and *L*_*n*_ ∼ 10 µm^[^ ^66^ ^]^. As discussed in Section 2.2, the extraordinary minority carrier diffusion length of SiC fundamentally changes the light‐trapping design of the PC, enabling much more facile fabrication than proposed for TiO_2_ photoelectrodes.^[^ ^33^, ^34^ ^]^

### 2.2 Suitable Photonic Crystal Structure

Photonic crystal architectures have been proposed for the enhancement of TiO_2_ PEC cell solar fuel generation efficiency^[^ ^33^ ^]^ and general photocatalysis efficiency.^[^ ^32^, ^34^ ^]^ The slanted conical‐pore PC with surface roughness exhibits the best light‐harvesting ability for TiO_2_ photoelectrode due to the synergy of built‐in antireflection, light‐trapping by slow‐light modes, branching of electron and hole transport, and the presence of straightforward charge carrier conduction paths.^[^ ^32^, ^34^ ^]^ Apart from the light‐trapping functionality, the morphology of the conical‐pore PC deliberately incorporates hierarchical length scales, thereby resolving the high recombination loss due to the small hole diffusion length in TiO_2_ and its strong mismatch with the electron diffusion length. On the one hand, the hole diffusion length (～10–300 nm^[^ ^62^, ^63^, ^64^, ^65^ ^]^) has to match half of the PC lattice constant (which is optimum at ∼150 nm), while on the other hand the thickness of the PC is limited to the electron diffusion length (∼10 µm^[^ ^66^ ^]^). Bearing this in mind, it is clear that for materials with small minority carrier diffusion lengths, the pore depth has to be roughly the same as the PC thickness to conform with the hierarchical length scales. For TiO_2_ this is still acceptable for fabrication as the thickness of the PC is limited to ≲ 10 µm.

The above architecture is not suitable for larger PC thicknesses due to the difficulty in manufacturing deep pores. This is exactly the case in 3C‐SiC with up to ∼200 µm majority carrier diffusion length. Fortunately, the minority carrier diffusion length of 3C‐SiC is also exceptionally long, up to ∼67 µm. This enables us to reduce the pore depth and increase the bulk thickness underneath the PC layer (see Figure 1a), allowing for facile fabrication by dry etching techniques.^[^ ^67^, ^68^ ^]^

In dry etching, the morphology of the pore is modified from cone to parabola. Such parabolic‐pore (Teepee PC) structure with periodicity 1.2–3.1 µm and depth ≳ 1.4 µm^[^ ^35^, ^67^, ^68^ ^]^ and similar inverted pyramid PCs of lattice constant 500 nm to 1.5 µm^[^ ^69^, ^70^ ^]^ have been fabricated on crystalline silicon by dry and wet etching methods, respectively. The slanted version of parabolic‐pore PC can be obtained by tilting the sample during the fabrication.^[^ ^36^ ^]^ A post‐treatment wet‐etch is also required to eliminate the large density of electronic defects induced by the dry‐etch. For SiC, the reactive ion etching, high‐temperature gas etching and wet etching are standard techniques,^[^ ^44^, ^71^ ^]^ and various silicon carbide PCs have been fabricated.^[^ ^72^, ^73^, ^74^ ^]^

The architecture of the photoelectrode is illustrated in Figure 1a. It consists of a surface protective coating (g‐CN, NiO, or 6H‐SiC) of thickness *d*_coat_, a 3C‐SiC PC composed of a (slanted) parabolic‐pore top layer on bulk unpatterned 3C‐SiC for a total 3C‐SiC thickness *d*_3C‐SiC_ ≡ *d*_PC_, and an SiO_2_ thin film rear passivation layer of thickness 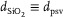 deposited on to a highly reflective metallic substrate. Periodic narrow metal protrusions on the substrate, separated by ∼*L*_*p*_ of 3C‐SiC, come into direct contact with the bulk 3C‐SiC and enable the photocurrent transport. The structure of the square‐lattice spbPore PC is characterized by lattice constant *a*, pore slant angle θ, inter‐pore mesa width *w*, an axial pore depth *h* measured along the pore axis (or at θ = 0), and a vertical pore depth *h*′ measured along the direction *z* (see Appendix F for detailed discussion).

The simulations of optical absorptivities, *A*, of the photoelectrode are carried out by solving Maxwell's wave equation for 45°‐polarized light (with respect to the *x*‐axis) at normal incidence, using the finite‐difference time‐domain (FDTD) method with spatial resolution 10 nm and Courant factor 0.5 (see Appendix G for more details). The required computing resources needed for FDTD simulations of weakly absorbing materials such as lightly doped 3C‐SiC quickly increase with the size and resolution of the unit cell, calling for accurate dielectric modeling and a reliable convergence analysis (see Appendices B.3 and G, respectively). The maximum achievable photocurrent density (MAPD) is taken to be the essential figure of merit to evaluate the light‐harvesting ability of the electrode:

(1) $$\begin{array}{c} \text{MAPD}=\frac{e}{hc}\int_{\lambda_{1}}^{\lambda_{2}}A\left(\lambda \right)I_{s}\left(\lambda \right)\lambda d\lambda \end{array}$$

Here *e* is the electron charge, *h* is the Planck constant, *c* is the speed of light in vacuum, *I*_*s*_ is the AM1.5G solar irradiance spectrum, and the spectral region [λ_1_, λ_2_] is taken as [280.5, 600] nm in this work.

## 3 Light Harvesting of 3C‐SiC Photonic Crystal Photocathode

We now optimize the 3C‐SiC spbPore PC structure without surface coating for light harvesting. In this case, photo‐excited electrons flow from 3C‐SiC into the aqueous solution above to produce hydrogen gas. A p‐type 3C‐SiC is assumed, which is considered stable for HER.^[^ ^14^, ^18^, ^20^, ^22^, ^75^ ^]^ This enables the photocathode application of the uncoated PC. Since we mainly focus on the photogeneration and charge transport processes, propitious cocatalysts deposition and surface porosity engineering are not explicitly included in the model. Moreover, p‐doped 3C‐SiC may greatly extend the absorption spectrum toward the longer wavelength region for visible light harvesting. This has been shown in boron‐doped 3C‐SiC to introduce an energy level 0.7 eV above the valence band.^[^ ^76^ ^]^

To facillate the photo‐excited electrons to flow from the bottom contact upward to the top 3C‐SiC‐solution interface, a bulk layer of p^+^‐type 6H‐SiC may be implemented between the bottom contact and the bulk 3C‐SiC to form type‐II heterojunction (see Section 4.3 for more details). 6H‐SiC shares the same chemical composition as 3C‐SiC. Their closely matched transverse lattice constants and nearly identical thermal expansion coefficients help minimize defect density at the interface,^[^ ^77^, ^78^ ^]^ making this heterojunction ideal for practical, out‐of‐lab solar‐driven water splitting applications. Such junction is routinely fabricated by pseudomorphic growth of 3C‐SiC along the [111] direction on top of on‐axis 6H‐SiC (0001) substrates.^[^ ^77^ ^]^ As outlined in Section 4.3, the complex refractive index for light with its electric field polarized perpendicular to the optical axis [0001] of 6H‐SiC is identical to that of 3C‐SiC.^[^ ^79^ ^]^ Therefore, when the 6H‐SiC layer lies deep beneath the 3C‐SiC, the dielectric models presented in Section 2.1 and Appendix B.1 can approximately describe the optical response of this heterojunction under normal incidence.

**Figure 2:**
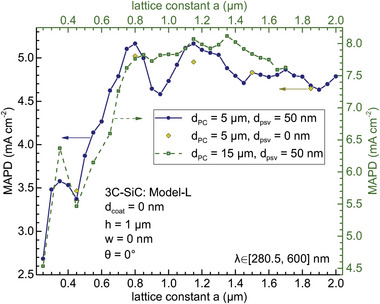
Light harvesting of square lattice normal parabolic‐pore PC in aqueous solution in the wavelength region of λ ∈ [280.5, 600] nm using the dielectric Model‐L. The MAPD as a function of the lattice constant *a*, for 5 µm‐ (blue solid circle and dark‐yellow diamond) and 15 µm‐thick (square) PCs with 1 µm pore depth, with (lines) and without (scatter) 50 nm‐thick SiO_2_ rear passivation layer.

In **Figure** **2** we investigate the effect of the PC lattice constant *a* on the light‐harvesting efficiency for 5 and 15 µm‐thick 3C‐SiC layers and 0 and 50 nm‐thick SiO_2_ rear passivation layers. This SiO_2_ layer is placed just below the 6H‐SiC electron‐blocking layer. The MAPD is optimized around *a* = 0.8 and 1.2 µm. Specifically, it reaches 5.16 mA cm^−2^ at *a* = 0.8 µm for 5 µm‐thick spbPore PC. The additional advantage of a smaller lattice constant is its larger specific surface area, which generally enhances the photocatalytic efficiency by exposing more reactive sites to the chemical reactants. Therefore, we primarily focus on the *a* = 0.8 µm case in the following. As shown in Figure 2, the rear passivation layer has a minor effect on the light harvesting. Nevertheless, it does improve light‐trapping and slightly increase the MAPD values around the two optimized lattice constants.

**Figure 3:**
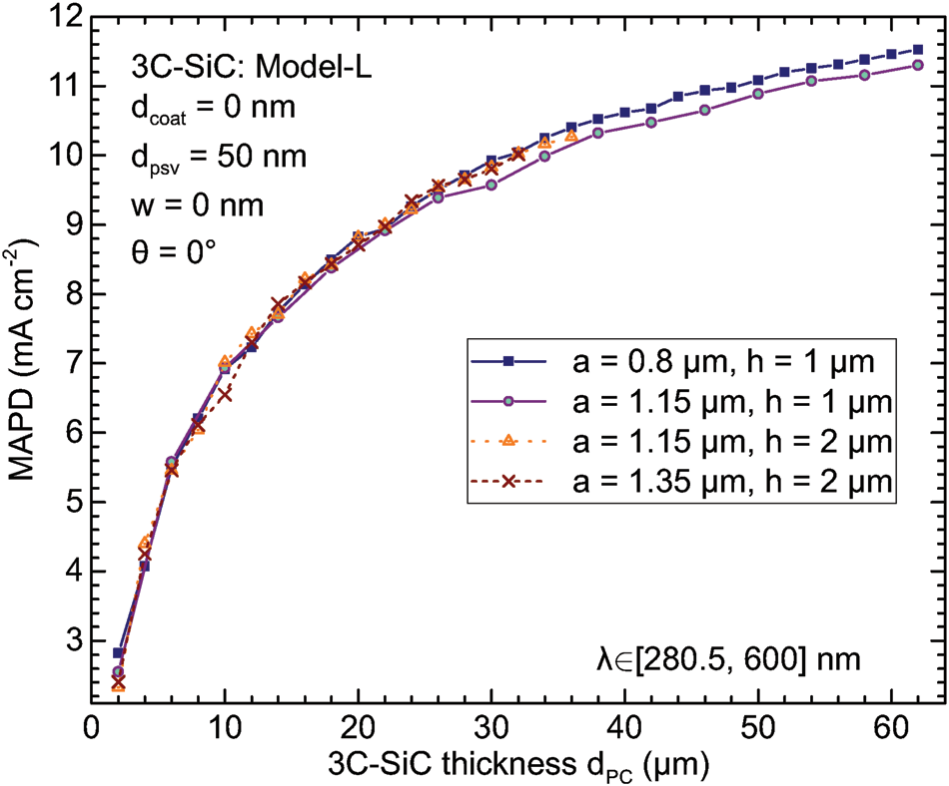
MAPD as a function of 3C‐SiC thickness *d*_PC_ for lattice constant of *a* = 0.8 µm (blue solid square), 1.15 µm (open circle and triangle), 1.35 µm (cross), and pore depth of *h* = 1 µm (solid lines), 2 µm (dashed lines), with a 50 nm‐thick SiO_2_ rear passivation layer.

**Figure 4:**
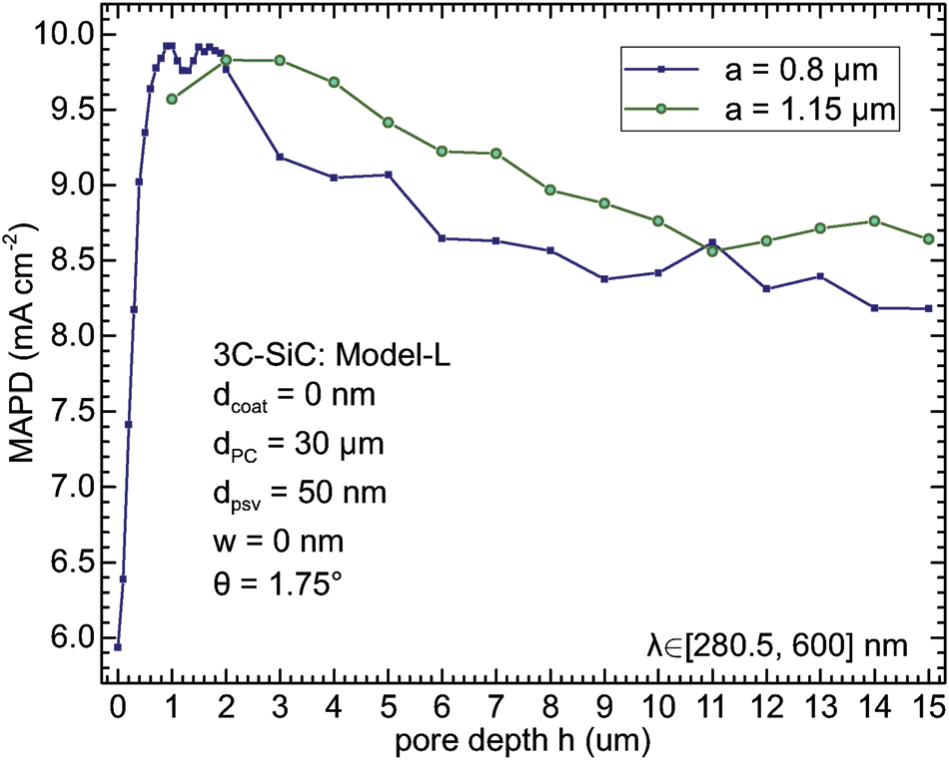
MAPD as a function of 3C‐SiC pore depth *h* from 0 to 15 µm for lattice constant *a* = 0.8 µm (navy blue) and 1.15 µm (green), and 3C‐SiC thickness 30 µm, with 50 nm‐thick SiO_2_ rear passivation layer.

In **Figure** **3** we fix three optimized values of the lattice constant shown in Figure 2, i.e., *a* = 0.8, 1.15, and 1.35 µm, and vary the thickness of 3C‐SiC *d*_PC_ from 2 to 62 µm, assuming pore depths of 1 and 2 µm. As discussed, the excellent transport property of 3C‐SiC enables the use of up to 55–67 µm‐thick PC for light harvesting. Figure 3 shows that for fixed pore depth *h* = 1 µm, the lattice constant *a* = 0.8 µm is slightly superior to *a* = 1.15 µm, while for fixed *a* = 1.15 µm, deeper pore of *h* = 2 µm results in better light‐trapping than *h* = 1 µm. At *h* = 2 µm, *a* = 1.15 µm on average performs better than *a* = 1.35 µm for thin PC with a thickness less than 15 µm, and no significant difference is seen for thicker PC. This suggests that the optimized combinations of the lattice constant and pore depth are (*a*, *h*) = (0.8, 1) µm and (1.15, 2) µm (see also **Figure** **4**). At *d*_PC_ = 30 µm, they demonstrate MAPD of 9.92 and 9.83 mA cm^−2^, respectively. Increasing the PC thickness to *d*_PC_ = 62 µm results in MAPD of 11.53 mA cm^−2^ for (*a*, *h*) = (0.8, 1) µm. In general, the MAPD experiences a steep rise with PC thickness for *d*_PC_ ≲ 20 µm because of the strong light‐trapping effect. The trend continues with a reduced slope due to absorption saturation. For *d*_PC_ ≳ 20 µm, shallow pore depth demonstrates higher MAPD due to the presence of more absorbing material, which is shown in Figure 4.

Figure 4 investigates the effect of the pore depth *h* on the light harvesting for the lattice constant *a* = 0.8 and 1.15 µm and 3C‐SiC thickness *d*_PC_ = 30 µm (in accordance with the conservative estimation of the minority carrier diffusion length of 29 µm discussed in Section 2.1). The optimized pore depth is a balance of the photonic light‐trapping and the reduction of the active photocatalytic material. Taking *a* = 0.8 µm as an example, as seen from Figure 4, a planar film (i.e., *h* = 0) demonstrates the least MAPD because of the absence of the photonic light‐trapping effect. As the pore becomes deeper, the MAPD rapidly increases from 5.93 mA cm^−2^ for *h* = 0 to 9.92 mA cm^−2^ for *h* = 1 µm. In this regime, the effective gradient refractive index reduces the reflection loss, and the densely distributed slow‐light modes in the formed PC structure result in strong light‐trapping in 3C‐SiC. This dramatically enhances the light absorptivity in the [280.5, 600] nm wavelength region and surpasses the effect of the decrease of the 3C‐SiC volume fraction. Furthering increasing the pore depth beyond 2 µm decreases the MAPD as the PC light‐trapping effect saturates and absorbing material reduces. For *a* = 1.15 µm, the optimized pore depth is ∼2–3 µm, agreeing with Figure 3. Considering the high hardness and inertness of SiC,^[^ ^9^ ^]^ a relatively shallow pore of *h* = 1 µm has advantages of superior light‐trapping and easier fabrication.

**Figure 5:**
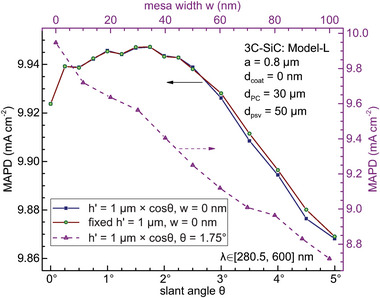
MAPD as a function of 3C‐SiC pore slant angle θ and mesa width *w* for lattice constant *a* = 0.8 µm, vertical pore depth *h*′ = 1 µm or *h*′ = 1 µm× cosθ and 3C‐SiC thickness *d*_PC_ = 30 µm, with 50 nm‐thick SiO_2_ rear passivation layer and no front coating layer.

**Figure F1:**
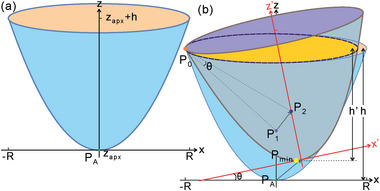
The model of the square lattice slanted parabolic‐pore PC. a) A normal parabolic‐pore with apex *P*_*A*_ (vertical coordinate *z*_apx_), base radius *R* and height *h*. b) The slanted parabolic‐pore obtained by rotating a normal parabolic‐pore by angle of θ in the *x*‐*z* plane around *y* axis with respect to a point *P*_0_. *h* and *h*′ are the pore depths of the normal and slanted parabolic pore, respectively.

It has been known that breaking the transverse rotation symmetry of the PC unit cell can enhance light‐trapping.^[^ ^28^, ^34^, ^36^ ^]^ In **Figure** **5** we tilt the parabolic pore to the *x* direction by a slant angle θ, pivoting at the point on the top edge in the *x*‐*z* plane through the vertical axis (see Figure F1). To better simulate the effect of the broken transverse symmetry on the unpolarized sunlight harvesting, we assume a source of 45°‐polarization (with respect to the *x*‐axis) of normal incidence. Since the vertical pore depth, *h*′, and the axial pore depth, *h*, are related by *h*′ ≈ *h*cosθ for small slant angles, the slant angle is varied in two ways: by fixing either *h*′ = 1 µm or *h* = 1 µm. It is worth noting that for θ ≠ 0, the physical pore depth corresponds to *h*′ rather than *h*, due to the location of the pivot point (see Appendix F for details). For *h* = 1 µm, the physical pore depth varies as *h*′ = 1 µm × cosθ. Figure 5 shows that the two give almost the same MAPD for slant angle θ ⩽ 2.25°, and only very minor difference up to θ of at least 5°. The optimized slant angle is θ = 1.75° where the MAPD is 9.947 mA cm^−2^ for *d*_PC_ = 30 µm. Further increasing θ reduces the light‐harvesting efficiency. Figure 5 also shows the degradation of the MAPD as the mesa width *w* increases from 0. Therefore, the mesa width is consistently taken as 0 throughout the paper, except in Figure 5.

**Figure 6:**
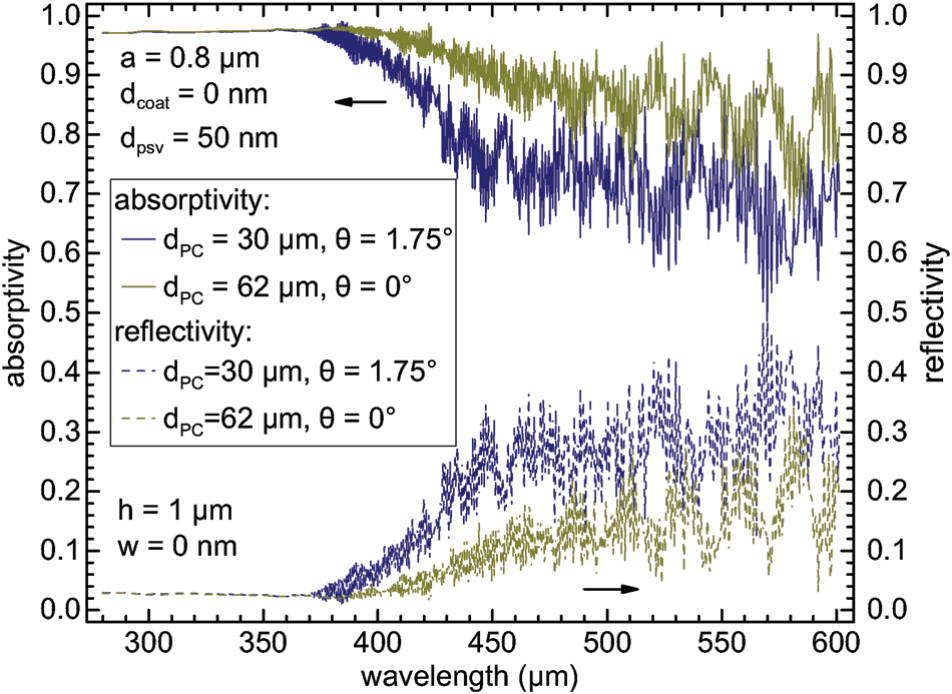
Absorptivity (solid lines) and reflectivity (dashed lines) spectra of 3C‐SiC slanted parabolic pore PC with dielectric Model‐L for 3C‐SiC thickness *d*_PC_ = 30 µm, slanted angle θ = 1.75° (navy blue lines), and *d*_PC_ = 62 µm, θ = 0° (dark yellow lines). Common parameters are lattice constant *a* = 0.8 µm, parallel slanted pore depth *h* = 1 µm, SiO_2_ rear passivation layer thickness *d*_psv_ = 50 nm and no front coating layer.

We have optimized the spbPore PC structure of lightly doped 3C‐SiC (with a very weak extinction coefficient) for light harvesting. Examples of absorptivity and reflectivity spectra for 30 and 62 µm‐thick spbPore PCs are given in **Figure** **6**, showing that PC light‐trapping enables more than 70% visible light absorptivity. The doping effects on the light absorption and charge transport are discussed in Appendix C.

## 4 Light Harvesting of 3C‐SiC Heterojunctions Photonic Crystal Photoanode

In this section, we explore the application of 3C‐SiC as a photoanode. In this case, a type‐II heterojunction between 3C‐SiC below and a thin coating layer above drives photo‐excited electrons to flow from the aqueous solution above, through the coating layer, into the 3C‐SiC region, and finally through the bottom contact to an external electrode in the solution. Anodic water oxidation imposes a much higher challenge to water splitting due to the slow four‐electron process.^[^ ^80^ ^]^ It also encounters severe instability issues on the electrode from the ubiquitous trade‐off between stability and activity.^[^ ^81^, ^82^ ^]^ So far the most stable and active oxygen evolution reaction (OER) electrocatalysts are iridium, ruthenium and their oxides.^[^ ^82^ ^]^ However, their extreme scarcities impose critical limits on the large‐scale deployment in electrochemical cells. The abundant SiC has excellent electronic properties and reinforced optical properties, enabled by PC architecture, for photoelectrodes. Whereas SiC is a durable photocathode material for reduction reactions,^[^ ^18^, ^21^ ^]^ it is prone to photocorrosion as a photoanode due to surface oxidation in OER.^[^ ^13^, ^17^, ^27^ ^]^ This calls for special measures to protect the 3C‐SiC photoanode, such as depositions of cocatalysts and an anti‐corrosion layer.

Here we discuss the enhanced photogeneration of 3C‐SiC spbPore PC by various absorptive, corrosion‐resistant surface coatings. Generally speaking, water oxidation and reduction are surface reactions, while photogeneration is largely a bulk process. Therefore, light‐trapping‐optimized 3C‐SiC PC can be used solely for optoelectronic purposes of photogeneration and transport, while relegating the chemical role to other more reactive, stable and/or cheaper photocatalysts.

While a protective coating on 3C‐SiC PC brings up interfacial losses of charge carriers, strategic engineering of the coating material and morphology can i) enhance the photogeneration due to antireflection effect (by the refractive index gradient) and/or additional light absorption (by the coating itself) and ii) reduce bulk carrier recombination by forming a type‐II semiconductor heterojunction (facilitating bipolar transport by advantageous relative band positions and a space charge region). Both p‐n 3C‐SiC homojunction^[^ ^22^ ^]^ and heterojunction (such as NiO/3C‐SiC^[^ ^23^ ^]^) have been applied to improve the PEC efficiency. Graphene coating on a 3C‐SiC photoanode has been shown to form a Schottky junction to facilitate carrier transport and protect the electrode from photocorrosion.^[^ ^83^ ^]^

### 4.1 g‐CN/3C‐SiC Heterojunction

In the past decade, there has been a new trend of shifting photocatalytic material from inorganic semiconductors to metal‐free organic materials such as graphitic carbon nitride^[^ ^84^, ^85^ ^]^ due to its cost effectiveness, environmental safety, tunability, and favorable band positions. Carbon nitride materials cover a broad class of derivatives,^[^ ^86^ ^]^ hereby collectively referred to as g‐CN, including the well‐known 1D polymer, melon, synthesized in 1834.^[^ ^87^ ^]^ Among these derivatives, the fully condensed forms with a stoichiometric composition of C_3_N_4_, a bandgap of ∼2.7 eV, and graphite‐like structures (g‐C_3_N_4_) — comprising linked s‐triazine (C_3_N_3_) or heptazine (tri‐s‐triazine, C_6_N_7_) rings — are considered the most stable allotrope under ambient conditions.^[^ ^88^ ^]^ The electron affinity of g‐C_3_N_4_ is ∼3.115 eV from density functional theory (DFT) calculation.^[^ ^89^ ^]^ Given the bandgap of ∼2.71 eV^[^ ^89^ ^]^, this results in a VBM of ∼−5.825 eV and a CBM of ∼−3.115 eV. The PZC for graphitic carbon nitride is ∼4–5,^[^ ^53^, ^90^ ^]^ and we adopt pH_PZC_ = 5.2^[^ ^53^ ^]^ in Figure 1b.

In spite of the advantages for applications in photocatalysis,^[^ ^91^ ^]^ the PEC reactivity of g‐CN remains low due to weak light absorptivity, low electrical conductivity, poor carrier separation efficiency, and relatively slow water‐oxidation kinetics.^[^ ^92^, ^93^ ^]^ A variety of methods have been implemented to improve the g‐CN PEC efficiency, including doping with different heteroatoms and molecules, increasing surface areas by porous structures and exfoliated nanosheets, and creating heterojunctions with other semiconductors.^[^ ^92^, ^94^, ^95^ ^]^ However, there has been no attempt to incorporate an efficacious light‐trapping mechanism to enhance its photogeneration. Optically, the light‐trapping in PCs relies on the refractive index contrast between the dielectric and the surrounding environment (aqueous electrolyte in PEC cell). But g‐CN has refractive index *n*_g‐CN_ ∼ 1.7,^[^ ^96^ ^]^ close to that of water 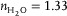. As a result, even fabricated into a PC morphology, g‐CN by itself does not trap light effectively. However, light‐trapping in g‐CN electrode can be achieved by its coating on a PC scaffold of high refractive index material, such as SiC with *n*_3C‐SiC_ ∼ 2.6–2.7.^[^ ^97^ ^]^ In this way, the g‐CN coating benefits from the lighttrapping of the overall structure, and its photogeneration rate is enhanced by the underlying PC. Electronically, as discussed in Section 2, both 3C‐SiC and g‐CN have conduction and valence bands positions straddling the redox potentials of water‐splitting, and they together form a type‐II heterojunction (Figure 1b), drawing holes to the surface of g‐CN and pushing electrons to the opposite terminal of 3C‐SiC. A more in‐depth discussion on the feasibility of the g‐CN/3C‐SiC heterojunction photonic crystal photoanode is found in Appendix D.

**Figure 7:**
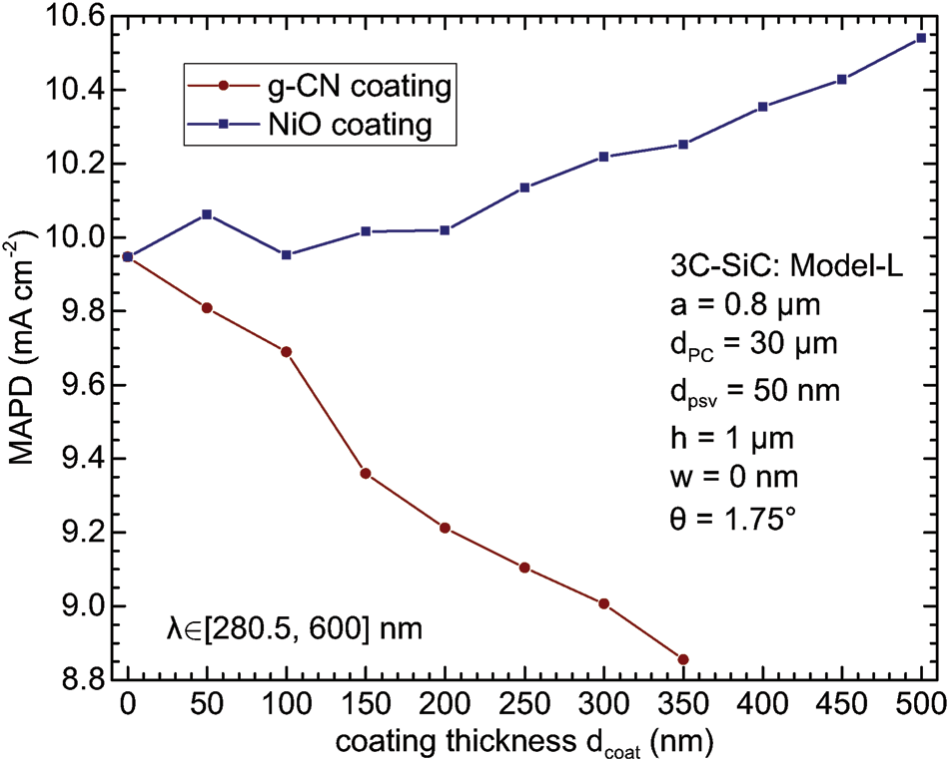
MAPD as a function of g‐CN and NiO coating thicknesses on optimized 3C‐SiC slant parabolic pore PC with Model‐L.

In **Figure** **7** we consider the photoanode consisting of a g‐CN top protective coating layer on the optimized 3C‐SiC spbPore PC discussed in Section 3. We assume uniform coating thickness along the pore axis direction (i.e., non‐conformal coating). The MAPD decreases as the protective layer thickness increases from 0 to 350 nm, in part due to the strong mismatch of the refractive indices of g‐CN and 3C‐SiC, and the fact that the underlying 3C‐SiC spbPore PC is already optimized for light trapping in aqueous solution. Nevertheless, for the coating layer thickness of 50 nm that we consider (see Appendix D for discussion), the MAPD still reaches as high as 9.81 mA cm^−2^ for *d*_PC_ = 30 µm, a reduction of only 1.4% with respect to the optimized MAPD of 9.95 mA cm^−2^ with no protective coating. Given the formation of the corrosion‐resistant layer and the type‐II heterojunction, and actual overall performance of this composite photoanode is superior to the non‐coated PC and traditional planar photoanodes. Experimental evidence can be found in recent work of g‐CN/3C‐SiC nanocomposite on visible light photocatalytic reactivity.^[^ ^98^ ^]^ In essence, g‐CN and 3C‐SiC spbPore PC can mutually benefit by forming a heterojunction.

### 4.2 NiO/3C‐SiC Heterojunction

The high MAPD (≳ 10 mA cm^−2^) achieved in the spbPore PC architecture imposes greater challenges to the durability of the photoelectrode. For high photocatalytic activity, ensuring the stability of the surface coating material is crucial.^[^ ^82^ ^]^ In basic environments, first‐row transition metal oxides such as NiO demonstrate remarkable durability and have been used as anti‐corrosion coatings on unstable semiconductor photoanodes.^[^ ^99^, ^100^, ^101^, ^102^, ^103^ ^]^

**Figure B2:**
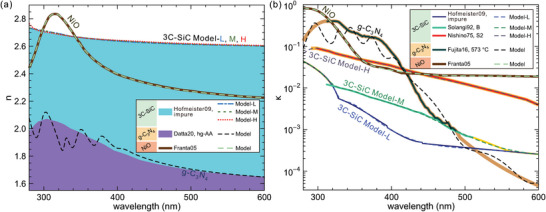
Dielectric models of lightly doped (Model‐L), moderately doped (Model‐M), and highly doped (Model‐H) 3C‐SiC, and of g‐CN (or specifically, g‐C_3_N_4_) and NiO, in the spectral region of λ ∈ [280, 600] nm. Notations of the data sets are the same as in Figure B1. a) The cyan filling, medium purple filling, and walnut solid line correspond to the refractive indices of experimental measurement on impure 3C‐SiC in ref. [79], DFT calculation on AA‐stacked heptazine g‐C_3_N_4_ in ref. [89], and experimental measurement on thin‐film NiO in ref. [113]. b) The navy blue, caribbean green, red, black and walnut solid lines correspond to the extinction coefficients of experimental measurements on impure 3C‐SiC in ref. [79], 3C‐SiC Sample B in ref. [138], 3C‐SiC Sample S2 in ref. [135], g‐C_3_N_4_ prepared under 573 °C substrate temperature in ref. [142], and thin‐film NiO in ref. [113], respectively. The highlighted light orange, light yellow, and light red‐brown thick solid lines correspond to extrapolated and/or complemented experimental data sets in order to build dielectric models in the entire 280 to 600 nm range. The dashed and dash‐dot lines in (a) and (b) are analytical curves fitted to the experimental and DFT data sets using the Lorentz model (B1) under the parameters specified in **Tables**  **B1**, **B2**, **B3** for 3C‐SiC, in **Table** **B4** for g‐C_3_N_4_, and in **Table** **B5** for NiO.

NiO has a bandgap *E*_*g*_ of 3.54–3.88 eV.^[^ ^101^, ^104^, ^105^, ^106^, ^107^ ^]^ In this work we take *E*_*g*_ = 3.7 eV, with band positions VBM = −5.4 eV and CBM = −1.7 eV (with respect to the vacuum level as shown in Figure 1b).^[^ ^108^, ^109^, ^110^ ^]^ Its PZC ranges from ∼8 to ≳ 10,^[^ ^51^, ^54^, ^111^, ^112^ ^]^ with a value of pH_PZC_ = 10.3^[^ ^54^ ^]^ adopted here. As shown in Figure 1b, NiO and 3C‐SiC form a type‐II heterojunction to facilitate charge carrier separation, with electrons flowing toward 3C‐SiC and holes flowing into NiO. The dielectric model of NiO in this work is based on experimental measurements of thin‐film samples in ref. [113] (see Table B5 and Figure B2 in Appendix  B.3).

Traditional NiO protective coatings involve a trade‐off between the transparency and conductivity mediated by tuning the hole density and film thickness of NiO.^[^ ^100^, ^101^, ^103^ ^]^ On the one hand, low transparency blocks the incident light from being harvested,^[^ ^100^ ^]^ partially accounting for the degradation of the photoelectrode.^[^ ^103^ ^]^ On the other hand, increasing transparency by reducing doping density and coating thickness may decrease the film conductivity and catalytic activity.^[^ ^101^, ^103^ ^]^ However, in our case, the NiO coating layer and the 3C‐SiC below form a type‐II heterojunction. In this case, the opacity of doped NiO is less of a hindrance since the photons absorbed in NiO can contribute useful photocurrent. This avoids the balancing problem of transparency and conductivity. Remarkably, as shown in Figure 7, rather than “blocking the incident light,” the MAPD of NiO‐coated 3C‐SiC spbPore PC can exceed that of the uncoated system. The feasibility of a NiO/3C‐SiC type‐II heterojunction photoanode has been confirmed experimentally for PEC water splitting.^[^ ^23^ ^]^ Further discussion on this feasibility is found in Appendix E.

In Figure 7, we investigate the influence of the NiO non‐conformal coating thickness (along the pore axis direction) on the MAPD, using the optimized 3C‐SiC spbPore PC discussed in Section 3. For coating thickness 50 nm, the MAPD of the NiO/3C‐SiC composite photoanode reaches 10.06 mA cm^−2^ for *d*_PC_ = 30 µm, boosted by 1.1% compared with the bare spbPore PC. Increasing the coating thickness to larger than 50 nm initially reduces MAPD until it reaches ∼150 nm, after which NiO begins to absorb strongly due to its much higher extinction coefficient (see Figure B2b). However, this thicker‐coating regime is accompanied by a high recombination loss of photo generated charge carriers, making it less favorable for optimal performance. Therefore, with a 50 nm coating thickness, the NiO/3C‐SiC spbPore photoanode simultaneously provides higher photogeneration, lower recombination loss, and better protection from corrosion.

### 4.3 6H‐SiC/3C‐SiC Heterojunction

Apart from g‐CN and NiO, the 6H polytype of SiC also holds promise as a coating material. The bandgap of 6H‐SiC is ∼3 eV,^[^ ^114^ ^]^ with a CBM ∼0.49^[^ ^115^ ^]^–0.55 eV^[^ ^114^, ^115^, ^116^, ^117^ ^]^ above that of 3C‐SiC, and a VBM ranging from 0.1 eV above to ∼0.11^[^ ^115^ ^]^–0.05 eV^[^ ^114^, ^116^, ^117^ ^]^ below that of 3C‐SiC. Due to the almost aligned VBMs, both type‐I and type‐II junctions have been reported for the 6H‐SiC/3C‐SiC interface.^[^ ^77^, ^114^, ^115^, ^118^ ^]^ Nevertheless, irrespective of the slight uncertainty in VBM offset, a proper doping (to form the appropriate p‐n junction) can drive electrons and holes in the desired directions for either the photocathode (see discussions in Section 3) or the photoanode application. In the latter case, this 6H‐SiC/3C‐SiC p‐n junction facilitates photogenerated electrons to flow from the liquid/6H‐SiC interface to the bulk 3C‐SiC and to a counter electrode, whereas photogenerated holes flow the opposite way. In the event that an SiO_2_ layer forms on top of 6H‐SiC during water splitting, this can be removed by HF etching, leaving behind surface hydroxyl groups.^[^ ^119^ ^]^ In the case of TiO_2_, such surface hydroxyl groups have proven useful in promoting liquid phase photocatalytic activity.^[^ ^120^ ^]^

6H‐SiC and 3C‐SiC can be epitaxially grown to form a high‐quality, stable heterojunction. Typically, this heterojunction is fabricated by growing 3C‐SiC (111) on 6H‐SiC (0001) substrates.^[^ ^77^, ^116^ ^]^ However, this fabrication approach is not compatible with coated PC structures, which requires the growth of 6H‐SiC on top of 3C‐SiC. The latter has been explored in some studies, such as the growth of 6H‐SiC (01$\bar{1}$4) planes on 3C‐SiC (001) substrates.^[^ ^77^, ^121^, ^122^, ^123^ ^]^

6H‐SiC is optically anisotropic due to its hexagonal crystalline structure. Its complex refractive index for the ordinary light, with electric field polarized perpendicular to crystal optical axis [0001], is almost the same as 3C‐SiC,^[^ ^79^ ^]^ while for the extraordinary light is different.^[^ ^124^ ^]^ However, given the small (∼50 nm) coating thickness of 6H‐SiC, the light‐trapping performance, MAPD of 6H‐SiC/3C‐SiC junction should be very close to that of 3C‐SiC alone analyzed in Section 3.

## 5 Conclusion

For half a century, photoelectrochemical (PEC) water splitting has been heralded as a promising avenue for transitioning from fossil fuels to clean, renewable energy. However, unlike the already industrialized silicon‐based photovoltaic technologies, solar light PEC water splitting has yet to see practical deployment, primarily due to persistent economic and environmental challenges. Despite ongoing efforts to improve water splitting efficiency — often under highly controlled conditions — advancements are frequently made at the expense of key factors such as cost‐effectiveness and environmental safety, which are essential for the realization of industrial‐scale applications.

This work aims to address these challenges by first selecting photocatalytic materials from a wide range of semiconductors, focusing exclusively on those that are cost‐effective, environmentally safe, and composed of abundant elements. These materials must also be suitable for high‐quality, high‐volume industrial production and demonstrate excellent charge transport along with moderate to high photoreactivity based on experimental evidences. To overcome the compromised sunlight photogeneration efficiency resulting from this restricted material selection, a carefully designed photoelectrode architecture is employed, incorporating a powerful photonic crystal light‐trapping mechanism.

Building on this strategy, we propose a 3C‐SiC slanted parabolic pore photonic crystal (PC) photocathode for the hydrogen evolution reaction, leveraging the remarkable cathodic stability of 3C‐SiC. The oxygen evolution reaction, however, presents a greater challenge due to its slower reaction kinetics and the susceptibility of the photoanode to photocorrosion. For this, we suggest a similarly structured photoanode coated with thin layers of g‐CN, NiO, or 6H‐SiC to form type‐II heterojunctions.

Optically, the 3C‐SiC PC provides a high‐index, light‐trapping, framework, while g‐CN and NiO coatings provide protection from corrosion and establish type‐II band alignments with 3C‐SiC. These coatings promote charge carrier separation and reduce recombination losses during carrier transport. Likewise, a thin 6H‐SiC layer, positioned either below or above 3C‐SiC, can enhance the crucial charge carrier separation for the photocathode or photoanode, respectively. In the latter case, an SiO_2_ layer forming on 6H‐SiC may require cleansing. Chemically, NiO exhibits remarkable stability, thereby protecting 3C‐SiC photoanode from photocorrosion. Although g‐CN has limited durability, its cost‐effectiveness and ease of synthesis make it a viable and appealing option for periodic re‐coating as a protective layer. In contrast to conventional light‐harvesting methods like heavy doping or quantum dot sensitization, this design simultaneously enhances both photogeneration and charge transport while improving chemical stability against corrosion, without compromising other critical factors.

We optimized a 3C‐SiC slanted parabolic pore PC on a highly reflective substrate for solar light trapping in the UV–vis wavelength range. A traditional 30 µm‐thick planar 3C‐SiC photocathode with light doping concentration ≲ 10^15^ cm^−3^ and a reflective back contact demonstrates an MAPD of 5.93 mA cm^−2^ in the wavelength region of [280.5, 600] nm. Experimentally, such a photoelectrode, featuring a p^+^ 4H‐SiC substrate beneath the 3C‐SiC layer and Pt cocatalyst on the surface, achieved a cathodic photocurrent of ∼−2.3 mA cm^−2^ at −0.5 V versus a saturated calomel electrode.^[^ ^21^, ^22^ ^]^ Our optimized 3C‐SiC photocathode has lattice constant of 0.8 µm, parallel pore depth 1 µm, slant angle 1.75° and SiO_2_ rear passivation layer 50 nm. For the same 30 µm‐thick, but optimized PC 3C‐SiC photocathode, the MAPD reaches 9.947 mA cm^−2^, representing 75.7% of the total available MAPD in this spectral range. That is 1.7 times the MAPD of the planar electrode, despite containing less 3C‐SiC, due to the exceptional light‐trapping capabilities of the photonic crystal slow‐light modes. The MAPD can be further increased to 11.102 mA cm^−2^ and 12.861 mA cm^−2^ for moderately and heavily doped 3C‐SiC, respectively, that is, 84.5% and 97.8% of the total available MAPD in the spectral region. Of these, the lightly doped 3C‐SiC has long enough carrier diffusion lengths to serve as a practical device.

With 50 nm‐thick coating layer and 30 µm‐thick 3C‐SiC, the g‐CN/3C‐SiC and NiO/3C‐SiC photoanodes demonstrate MAPDs of 9.808 mA cm^−2^ and 10.061 mA cm^−2^, respectively, in the case of lightly doped 3C‐SiC. These are 74.6% and 76.5% of the total available MAPD in this spectral range. Since the minority charge diffusion length can reach more than 60 µm, the MAPDs of both bare and coated PC can reach beyond 11.53 mA cm^−2^ in a 62 µm‐thick 3C‐SiC device.

This work describes both the opportunities and the challenges of utilizing an SiC photoelectrode. We hope that our results will help promote further experimental studies in the use of 3C‐SiC as a light‐trapping photoelectrode and the much needed investigation of protective coatings required to deter corrosion in aqueous environments.

## Conflict of Interest

The authors declare no conflict of interest.

## Figures and Tables

**Figure B1:**
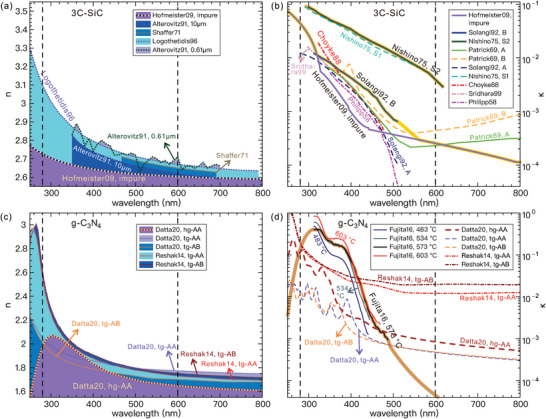
Experimental data on the refractive indices *n* (dotted lines with color fillings) and extinction coefficients κ (lines without color fillings) of a,b) 3C‐SiC and c,d) g‐C_3_N_4_. The vertical dashed lines demark the frequency range of interest, i.e., λ ∈ [280.5, 600] nm. a, b) Data sets of 3C‐SiC are denoted by author‐year notation followed by the sample name, i.e., “Hofmeister09, impure”: the “impure 3C SiC” in ref. [79]; “Alterovitz91, 10 µm(0.61 µm)”: the 10 µm(0.61 µm)‐thick sample in ref. [137]; “Shaffer71”: 3C‐SiC in ref. [134]; “Logothetidis96”: 3C‐SiC in ref. [139]; “Solangi92, A(B)”: Sample A(B) in ref. [138]; “Nishino75, S1(S2)”: Sample S1(S2) in ref. [135]; “Patrick69, A(B)”: Sample A(B) in ref. [133]; “Choyke88”: 3C‐SiC in ref. [136]; “Sridhara99”: 3C‐SiC in ref. [140]; “Philipp58”: 3C‐SiC in ref. [132]. Model‐L is based on *n* (navy blue‐sand yellow dashed line with medium purple color filling) and κ (medium purple solid line) of “Hofmeister09, impure”. Model‐M is based on *n* of “Hofmeister09, impure” and κ of “Solangi92, B” (blue solid line), extrapolated and complemented (deep yellow thick solid line) in part by κ of “Hofmeister09, impure”. Model‐H is based on *n* of “Hofmeister09, impure” and κ of “Nishino75, S2” (hunter green solid line), complemented (light orange thick solid line) in part by κ of “Nishino75, S1” (light green dashed line). c, d) Data sets of g‐C_3_N_4_ are denoted by author‐year notation followed by the structure or sample name, i.e., “Datta20, hg‐AA”: the DFT result of AA‐stacked heptazine g‐C_3_N_4_ in ref. [89]; “Datta20, tg‐AA(AB)”: the DFT result of AA(AB)‐stacked triazine g‐C_3_N_4_ in ref. [89]; “Reshak14, tg‐AA(AB)”: the DFT result of AA(AB)‐stacked triazine g‐C_3_N_4_ in ref. [141]; “Fujita16, 483(534, 573, 603) °C”: the sample prepared under the substrate temperature of 483(534, 573, 603) °C in ref. [142]. The Lorentz dielectric model of g‐C_3_N_4_ is based on *n* of “Datta20, hg‐AA” (red‐brown‐sand yellow dashed line with medium purple color filling) and κ of “Fujita16, 573 °C” (black solid line), extrapolated to the entire 280 to 600 nm spectral range (light red‐brown thick solid line). The DFT results are the average of three components of *n*(κ) for electric field polarizing along and perpendicular to the optical axis, which have similar values to that obtained from averaging three components of the dielectric constant. The extinction coefficients of “Datta20, h(t)g‐AA(AB)” presented here are from raw data of ref. [89] via private communication.

**Figure C1:**
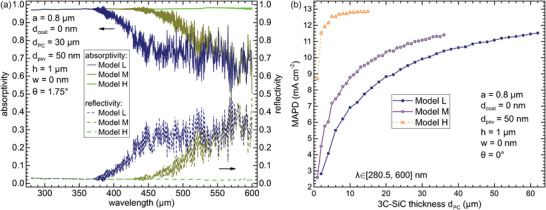
A light harvesting comparison of lightly doped (Model‐L), moderately doped (Model‐M), and highly doped (Model‐H) 3C‐SiC (slanted) parabolic pore PCs of lattice constant 0.8 µm, SiO_2_ rear passivation layer thickness 50 nm and no front coating layer. a) Absorptivity and reflectivity spectra for spbPore PC with slanted pore depth *h* = 1 µm, slant angle θ = 1.75°, and 3C‐SiC thickness *d*_PC_ = 30 µm. b) MAPD of spbPore PC with pore depth *h* = 1 µm and slant angle θ = 0°.

**Figure G1:**
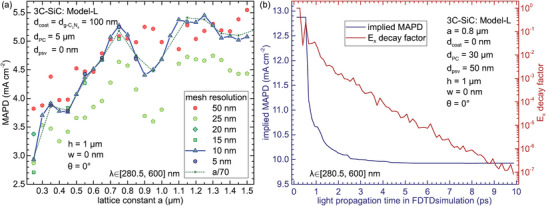
Convergence analysis of FDTD simulations. a) The MAPD as a function of the lattice constant *a* for various FDTD mesh resolutions for the simulation of the light harvesting of normal parabolic‐pore PC with *d*_PC_ = 5 µm‐thick 3C‐SiC, $d_{\mathrm{coat}}=100$ nm‐thick g‐CN coating, *h* = 1 µm pore depth and no rear passivation layer. b) The implied MAPD and *E*_*x*_ decay factor as functions of physical light propagation time. The simulated structure is a normal parabolic‐pore PC of 0.8 µm lattice constant, on a highly reflective substrate in aqueous environment, with 30 µm‐thick 3C‐SiC (dielectric Model‐L), 1 µm pore depth, 50 nm SiO_2_ rear passivation and no surface coating.

## Data Availability

The data that support the findings of this study are available from the corresponding author upon reasonable request.

## Appendix A Transport Properties

3C‐SiC has a conduction band at −3.83 eV and a valence band at −6.18 eV relative to the vacuum of empty space and at pH_PZC_, allowing them to straddle the H^+^/H_2_ reaction level at −4.44 eV and the O_2_/H_2_O reaction level at −5.67 eV in solutions at pH = 0,^[^ ^45^ ^]^ as illustrated in Figure 1b. 3C‐SiC single crystals have been fabricated using heteroepitaxial methods^[^ ^71^, ^125^ ^]^ on silicon and silicon carbide substrates, with sample quality continuously improving to a very high level in recent years.^[^ ^59^, ^60^, ^126^ ^]^.

3C‐SiC has a resistivity of ρ ∼ 18 Ω cm for carrier density 2 × 10^16^ cm^−3^.^[^ ^76^ ^]^ The hole (Hall) mobility is ∼40–100 cm^2^ V^−1^ s^−1^^[^ ^44^, ^71^ ^]^ and the electron (Hall) mobility is ∼500–1000 cm^2^ V^−1^ s^−1^^[^ ^44^, ^61^, ^127^ ^]^ for carrier density ∼10^16^ cm^−3^, corresponding to diffusion coefficients of holes *D*_*p*_ ∼ 1.0–2.6 cm^2^ s^−1^ and electrons *D*_*n*_ ∼ 12.8–25.7 cm^2^ s^−1^, respectively. The ambipolar diffusion coefficient is ∼3–4 cm^2^ s^−1^,^[^ ^60^, ^128^ ^]^ from which the extracted hole diffusion coefficient ∼2.17 cm^2^ s^−1^ and electron diffusion coefficient ∼19.5 cm^2^ s^−1^^[^ ^60^ ^]^ agree with values from the Hall measurements. The photogenerated charge carrier lifetime for n‐type 3C‐SiC ranges from ≲ 50 ns up to 8.2 µs at doping density ∼10^15^ cm^−3^.^[^ ^59^, ^60^, ^129^ ^]^ The bulk carrier lifetime was estimated to reach as high as τ_*B*_ = 10–15 µs^[^ ^59^ ^]^ (by assuming *D*_*p*_ = 3 cm^2^ s^−1^), and the surface recombination velocity is *S* = 300–10^4^ cm s^−1^,^[^ ^59^, ^129^ ^]^ similar to that of 4H‐SiC (1000–7500 cm s^−1^) at the same doping level (∼10^15^ cm^−3^).^[^ ^130^, ^131^ ^]^

Therefore, for a properly passivated 3C‐SiC photoelectrode, the minority carrier (hole) diffusion length, $L_{p}=\sqrt{D_{p}\tau_{B}}$, can reach the exceptional scale of 55–67 µm. Likewise, the majority carrier (electron) diffusion length is as large as *L*_*n*_ ∼ 165–201 µm (assuming *D*_*p*_ = 3 cm^2^ s^−1^ and *D*_*n*_ = 9*D*_*p*_). Even considering a much smaller *D*_*p*_ = 1 cm^2^ s^−1^ and unpassivated carrier lifetime 8.2 µs, the hole diffusion length still reaches *L*_*p*_ ∼ 29 µm.

## Appendix B Optical Properties and Dielectric Models

### B.1 Optical Properties of 3C‐SiC

The as‐grown 3C‐SiC is an n‐type semiconductor due to unintentional nitrogen doping. Its absorption coefficient is highly sensitive to this doping concentration, necessitating careful analysis. The optical properties of 3C‐SiC single crystal have been measured by numerous groups over the past five decades.^[^ ^79^, ^132^, ^133^, ^134^, ^135^, ^136^, ^137^, ^138^, ^139^, ^140^ ^]^ These experimental studies are presented for comparison in **Figure** **B1**a,b in terms of refractive index (or the real part of the complex refractive index) *n* ^[^ ^79^, ^134^, ^137^, ^139^ ^]^ and extinction coefficient (or the imaginary part of the complex refractive index) κ,^[^ ^79^, ^132^, ^133^, ^135^, ^136^, ^138^, ^140^ ^]^ showing up to two orders of magnitude variation in κ, presumably due to differences in doping levels and defects densities.^[^ ^138^ ^]^ To account for this material variation, we built three distinct dielectric models based on these experiments: Model‐L, Model‐M, and Model‐H, corresponding to lightly, moderately, and heavily doped 3C‐SiC single crystals, respectively.

Model‐L for lightly doped and/or pristine 3C‐SiC is based on the dielectric data of the “impure 3C‐SiC” in ref. [79]. The dielectric function reported in ref. [79] is a hybrid of measurements and interpolations in different spectral ranges, giving rise to broadband data of the refractive index *n* and the extinction coefficient κ (see Figure B1a,b) for high‐quality sample with low doping concentration (supposedly ∼10^15^ cm^−3^). This dielectric function is adopted because it enables the use of an established optical model, especially for the refractive index, without the need for additional data extrapolation. The refractive indices of the two sets of dielectric functions in ref. [79], for “pure” and “impure” 3C‐SiC, respectively, have almost the same values, which is expected as the refractive index is less dependent on the doping level. Thus we adopt the “impure” refractive index data from ref. [79] for all three dielectric models, Model‐L, ‐M, and ‐H, of 3C‐SiC. The absorption coefficient is mostly based on measurements of the ordinary ray, light with the electric field component perpendicular to the crystal optical axis, on 6H‐SiC, but is reported to be applicable to 3C‐SiC.^[^ ^79^ ^]^ The latter does not exhibit birefringence due to its cubic crystal structure. Nevertheless, as seen from Figure B1b, the “impure 3C‐SiC” dielectric function in ref. [79] generally represents the lower bound of the extinction coefficient κ among the ten selected datasets. Therefore, it is used to model a slightly doped 3C‐SiC single crystal in this work.

Model‐M is derived from the absorption coefficient α = 4πκ/λ of the 10.7 µm‐thick 3C‐SiC single crystal “Sample B” grown heteroepitaxially on (100) Si substrate using chemical vapor deposition with 20 nm buffer layer, as described in ref. [138]. The sample has a carrier concentration of 6.9 × 10^16^ cm^−3^, making it suitable for modeling a moderate‐quality 3C‐SiC single crystal with some fabrication defects. However, the absorption and extinction coefficients data of this sample is available only within a limited spectral range of ∼[311.1, 511.1] nm.^[^ ^138^ ^]^ To extend the data, we extrapolate the extinction coefficient from 511.2  to 562 nm using an exponential fitting function κ = exp (− 22.0931 + 10.7811*f* − 1.65128*f* ^2^) where *f* = 1/λ (µm^−1^). Additionally, we supplement the data for λ ⩽ 319.5 nm and λ ⩾ 561.8 nm by the extinction coefficient of the impure sample in ref. [79] (see Figure B1b). The refractive index is sourced from the impure sample in ref. [79] and thus requires no further extension.

Model‐H is based on the absorption coefficient of 3C‐SiC single crystal film reported in ref. [135], epitaxially grown on (111) Si substrate without a buffer layer.^[^ ^138^ ^]^ The film has a thickness of ∼1 µm and a carrier concentration of ∼10^19^ cm^−3^. The samples are expected to have a higher defect density compared with that used in Model‐M,^[^ ^138^ ^]^ rendering Model‐H appropriate for describing low‐quality 3C‐SiC with high doping level and large amounts of structural defects. Data from “Sample S2” (λ ∈ [298.1, 618.7] nm) was adopted and supplemented with an additional data point from “Sample S1” at λ = 290.6 nm, extending the short wavelength limit from 298.1  to 290.6 nm. The refractive index was taken from the impure sample in ref. [79].

Comprehensive numerical details of these three dielectric models can be found in Appendix B.3. A comparison of their inferred absorption spectra, along with a discussion on their suitability for PC light‐trapping, is provided in Appendix C.

### B.2 Optical Properties of g‐CN

Up to now, accurate measurement on the optical properties of g‐CN is scarce,^[^ ^142^ ^]^ and the refractive index dispersion has not been experimentally reported. Fortunately, density functional theory (DFT) shines some light on the dielectric function of g‐C_3_N_4_ from the theoretical point of view.^[^ ^89^, ^141^ ^]^ In Figure B1c,d we compare the experimental and theoretical data on g‐C_3_N_4_ refractive index *n* and extinction coefficient κ.

Our dielectric model of g‐CN is based on the absorption coefficient measurement of the stoichiometric sample prepared at 573 °C substrate temperature (Sample 573°C) in ref. [142], and the refractive index calculation of the heptazine g‐C_3_N_4_ polymorph from DFT simulation in ref. [89].

The experimental data of the absorption coefficient in ref. [142] is valid in the region of λ ∈ [311.5, 488.1] nm. To access the optical property of the whole spectral range of interest, λ ∈ [280.5, 600] nm, we extrapolate the long‐wavelength extinction coefficient from 488.1  to 600 nm by an exponential fitting function κ = exp (− 12.04921 − 3.84427*f* + 3.03725*f* ^2^), and the short‐wavelength extinction coefficient from 311.5  to 282 nm by an exponential fitting function κ = exp (− 72.83311 + 45.25422*f* − 7.11731*f* ^2^), where *f* = 1/λ (µm^−1^) (see Figure B1d). Since the extrapolated [488.1, 600] nm spectral region assumes low extinction coefficient ∼10^−5^–10^−4^, and [282, 311.5] nm spectral region has weak solar irradiation, the influence of the extrapolated part of the optical model is insignificant.

The refractive index *n* of g‐C_3_N_4_ is based on its DFT calculation of AA‐stacked heptazine polymorph (h‐g‐C_3_N_4_) in ref. [89]. The major motivation for the adoption of h‐g‐C_3_N_4_ (among heptazine and triazine polymorphs investigated in ref. [89]) is its favorable theoretical band positions (reported therein) with respect to the oxidation/reduction potentials for water splitting, and with respect to those of 3C‐SiC^[^ ^45^, ^47^ ^]^ for forming a type‐II heterojunction. It is worth noting that triazine g‐C_3_N_4_ of AA and AB stacks may also demonstrate favorable bands positions according to other DFT calculations such as in ref. [143]. The refractive index of g‐C_3_N_4_ was measured to be 1.77 at 514.5 nm in ref. [96], which is reasonably close to the value of 1.7 of h‐g‐C_3_N_4_ in ref. [89] (see Figure B1c).

### B.3 Dielectric Models of 3C‐SiC, g‐CN and NiO

In this work, optical properties of 3C‐SiC, g‐CN and NiO are described by a Lorentz model of the dielectric function

(B1)
$$\begin{array}{c} \varepsilon \left(\omega \right)=\varepsilon_{\infty }+\sum_{n}\frac{s_{n}\omega_{n}^{2}}{\omega_{n}^{2}-\omega^{2}-i\omega \gamma_{n}} \end{array}$$

where ε = ε′ + *i*ε″, ω is the frequency of incident light, ε_∞_ is the dielectric constant in the limit of infinite frequency, ω_*n*_ and γ_*n*_ are the resonance frequency and width of the *n*th Lorentz oscillator, respectively, and *s*_*n*_ specifies the strength of the *n*th resonance.

It is worth noting that the extinction coefficient, related to the imaginary part of the dielectric function (B1), for weakly absorbing materials such as lightly doped 3C‐SiC typically spans more than 3 orders of magnitude from the UV to the visible frequency range and falls below ≲ 10^−3^. Consequently, an imprecise fit can easily have negative values in or outside the spectral range of interest ([280.5, 600] nm wavelength in our case). Such negative contribution, even outside the spectral region of interest, regardless of its relative smallness, can quickly lead to an instability of the FDTD simulation for weakly absorbing materials. Therefore, dielectric models of weakly absorbing materials need to be carefully fitted to avoid poles in the entire spectral range beyond the frequency region of interest.

The fitting parameters of 3C‐SiC Model‐L, ‐M, and ‐H are given in **Table** **B1**, **B2**, and **B3**, respectively. The fitting parameters of g‐CN and NiO are given in Table **B4** and Table **B5**, respectively. In **Figure** **B2** we compare the analytical Lorentz models with the (extrapolated and complemented) experimental data.

**Table B1:** Fitting parameters of the dielectric function for lightly doped single crystalline 3C‐SiC (Model‐L) using Lorentz model (B1) based on the dielectric data of the impure sample in ref. [79].

*n*	*s*_*n*_	ω_*n*_ [µm^−1^]	γ_*n*_ [µm^−1^]
1	0.19509693828	4.471527638599	2.132102877404
2	0.48502136586	5.731388649134	6.700418× 10^−11^
3	−0.02845151474	2.746111621347	2.287740750284
4	0.06587621789	4.803405294359	0.790953223582
5	−0.03928080402	3.045866135128	1.445659550814
6	−0.00288416340	3.053428429269	0.265270793231
7	0.33940945234	5.739172629831	1.734087× 10^−9^
8	−0.00975251113	1.810688936307	2.407457504842
9	0.49999999986	0.212867178882	1.208816× 10^−28^
ε_∞_	5.66986625977372		

**Table B2:** Fitting parameters of the dielectric function for moderately doped single crystalline 3C‐SiC (Model‐M) using Lorentz model (B1) based on the refractive index of the impure sample in ref. [79] and absorption coefficient of Sample B in ref. [138].

*n*	*s*_*n*_	ω_*n*_ [µm^−1^]	γ_*n*_ [µm^−1^]
1	−0.00518429766	3.137755139035	0.391562063447
2	0.04077096144	0.040149290965	2.240721511135
3	−0.02137646625	2.371569457933	1.683695206569
4	1.05957560990	6.113361188193	0.117926172222
5	2.22222222222	0.076502235102	2.280523× 10^−13^
6	−0.00031345643	0.993562218295	3.999999995493
7	0.06034320720	4.149116217315	1.486487418621
8	0.18739791871	5.048211230455	1.489255010186
9	0.01150471752	1.701059896712	1.397144640764
10	−0.02019980977	2.977172346846	1.350382083963
11	−0.00133756867	0.881379392443	1.200286395148
12	0.00145847021	2.398785177324	0.620393974365
13	−0.02184016911	1.741944487918	1.685217047893
14	6.531272× 10^−8^	0.400000299166	3.070378 × 10^−11^
15	2.22222222222	0.076492998846	1.826628× 10^−11^
ε_∞_	5.40915373503592		

**Table B3:** Fitting parameters of the dielectric function for highly doped single crystalline 3C‐SiC (Model‐H) using Lorentz model (B1) based on the refractive index of the impure sample in ref. [79] and absorption coefficient of Sample S2 in ref. [135].

*n*	*s*_*n*_	ω_*n*_ [µm^−1^]	γ_*n*_ [µm^−1^]
1	−0.00555555553	1.626695981700	0.439546653878
2	0.04956543151	3.418336218691	0.562748531658
3	0.11111111093	3.988623749311	1.062681× 10^−8^
4	0.11111111104	3.991544066600	3.801172× 10^−8^
5	0.11111110231	3.987269976924	1.213663× 10^−9^
6	0.07458421281	3.186906586362	1.129550237785
7	0.01847121592	2.616461827010	0.762164900183
ε_∞_	6.18801570363816		

**Table B4:** Fitting parameters of the dielectric function for g‐C_3_N_4_ using Lorentz model (B1) based on the density functional theory calculation of the refractive index of AA‐stacked heptazine g‐C_3_N_4_ in ref. [89] and the absorption coefficient of the sample 573 °C in ref. [142].

*n*	*s*_*n*_	ω_*n*_ [µm^−1^]	γ_*n*_ [µm^−1^]
1	0.00503051879	2.479565820634	0.115367122143
2	0.02203307089	2.677850270429	0.116918115222
3	0.05040000000	2.901571465700	0.154818934777
4	0.01440000000	3.046772449619	0.072902027603
5	0.09748767445	3.375000483309	0.227985096263
6	1.19159210575	4.255512969452	7.2500744× 10^−11^
7	−0.00354990300	1.787700000000	0.812967966337
8	−0.00393097600	2.121946245758	0.454416683108
ε_∞_	1.05000001776913		

**Table B5:** Fitting parameters of the dielectric function for NiO using Lorentz model (B1) based on the thin‐film sample in ref. [113].

*n*	*s*_*n*_	ω_*n*_ [µm^−1^]	γ_*n*_ [µm^−1^]
1	1.512607580659	5.393987033816	2.453614632788
2	0.079938884991	4.289807101735	0.471880883613
3	−0.33333333332	2.816619681892	2.126552493012
4	0.800158673433	3.392528485489	0.736931672875
5	0.166241083602	3.888727967590	0.570542664476
6	0.750593293821	6.675717056138	2.0128773× 10^−8^
7	−0.33333333333	3.008023881586	0.779410738914
ε_∞_	1.99544765916791		

Since the light harvesting primarily occurs in the 3C‐SiC part of the PC, it is important to accurately model the 3C‐SiC dielectric function. From Figure B2, the Lorentz models of 3C‐SiC, as well as NiO, fit the (extrapolated and complemented) experimental data very well for λ ∈ [280, 600] nm. The Lorentz model of g‐CN, on the other hand, deviates from the designated values of the refractive index and extinction coefficient in a few spectral regions. Numerically, this arises from a compromise between fitting accuracy and the necessity to maintain the positivity of the dielectric function's imaginary part across a broader spectral range, ensuring the stability of the FDTD simulation. Physically, we make no attempt to achieve very high fitting accuracy for g‐CN because: 1) The major source of the uncertainty comes from the hybrid DFT‐experiment dielectric data, which could cause deviation from the actual dielectric function much more than that in the Lorentz fitting procedure; 2) Actual g‐CN contains a rich class of derivatives with widely variable optical properties; 3) The extinction coefficient of g‐CN is small in the visible frequency regime due to its large bandgap, and the thickness of the g‐CN coating is much thinner than the 3C‐SiC layer of the PC. As a result, the inaccuracy of the dielectric model of g‐CN has only minor effect on the light‐harvesting efficiency.

## Appendix C Optical Implications of “Lightly Doped” 3C‐SiC

The optical absorptivity of our spbPore PC depends on the doping level of 3C‐SiC, described by three different dielectric functions Model‐L, ‐M and ‐H. Our PC photoelectrode architecture is ideally suited for lightly doped 3C‐SiC as described by Model‐L, which offers a balance between low electronic transport loss and high photogeneration gain. This synergy is achieved through the material's low‐defect concentration and high crystallinity, and the strong light‐trapping capabilities of our photonic crystal architecture.

Apart from the inferred carrier concentrations given in Appendix B.1, it is useful to understand the optical implications of being “lightly doped.” This appendix clarifies this issue by comparing the MAPD of our three dielectric models of 3C‐SiC.

In **Figure** **C1**a, we plot the absorptivity and reflectivity spectra for three dielectric models of 3C‐SiC with already optimized structural parameters for *d*_PC_ = 30 µm. The MAPDs in the wavelength region of [280.5, 600] nm reach 9.95, 11.10, and 12.86 mA cm^−2^ for Model‐L, ‐M, and ‐H, respectively. These represent 75.7%, 84.5%, and 97.8% of the total available MAPD in this spectral range. For Model‐L, the minority carrier diffusion length *L*_*p*_ may be longer than 30 µm. Therefore even more MAPD than 9.95 mA cm^−2^ can be achieved by using a thicker PC cell.

A direct comparison of the MAPD under the three models as a function of the PC thickness is given in Figure C1b. However, it is important to note that for Model‐H in this comparison, the value of *L*_*p*_ will be orders of magnitude smaller than 30 µm. Consequently, the actual photocurrent density will be considerably lower than 12.86 mA cm^−2^ for *d*_PC_ = 30 µm, primarily due to recombination losses. Similar considerations apply to Model‐M. In fact, it is known from Si‐based photovoltaics that a doping level of 10^19^ cm^−3^ in Model‐H typically results in a very short diffusion length of only a few hundred nanometers, imposing severe restrictions on the thickness of the photoelectrode for effective PC light‐trapping.

## Appendix D Feasibility of g‐CN/3C‐SiC Heterojunction Photonic Crystal Photoanode

Recently, graphitic carbon nitride (g‐CN) has attracted significant research interest as a novel metal‐free photoelectrode semiconductor. Carbon nitride materials are composed of C, N and residual H. Both carbon and nitrogen are abundant, cost‐effective, and environmentally friendly. Experimentally, partially condensed 2D crystalline carbon nitrides, poly(heptazine imide)^[^ ^144^ ^]^ and poly(triazine imide)^[^ ^145^ ^]^ were prepared, and crystalline films of triazine‐based graphitic carbon nitride have been interfacially synthesized, reporting a bandgap of 1.6–2.0 eV.^[^ ^146^ ^]^ These 2D carbon nitrides, sometimes including melon, may have been all loosely called g‐C_3_N_4_.^[^ ^85^ ^]^ In this work, g‐C_3_N_4_ refers to the stoichiometric graphitic carbon nitride, and g‐CN to the general g‐C_3_N_4_‐like derivatives. Nevertheless, carbon nitrides synthesized today are usually in intermediate condensation stages with a fair amount of hydrogen and other defects and/or dopants. The electron diffusion length of compact polycrystalline g‐CN film is on the order of 1 µm.^[^ ^147^ ^]^

Graphitic carbon nitride is an actively studied functional material for fuel cell catalyst support,^[^ ^148^ ^]^ metallic nanoparticle synthesis,^[^ ^94^ ^]^ luminescence and electrochemical sensing,^[^ ^149^ ^]^ and photocatalysis for hazardous pollutant decomposition, CO_2_ reduction and water splitting.^[^ ^91^, ^92^, ^94^, ^95^ ^]^

It is worth noting that so far carbon nitride photoanode still suffers from chemical instability due to self‐oxidation.^[^ ^92^, ^150^ ^]^ However, the stability is continuously improving by incorporating cocatalysts. Recent Ni‐Fe based metal‐organic framework, integrated with reduced graphene oxide, enhances the carrier diffusion and hole extraction efficiencies, thereby extending the stability time to 35 h. This might be further increased by fixing the slow leaching of the catalyst.^[^ ^93^ ^]^ On the other hand, protective coatings made from inexpensive materials with limited long‐term stability present an alternative pathway for commercialization.^[^ ^92^ ^]^ Despite the abundance of 3C‐SiC, producing high‐quality single‐crystal PC still significantly contributes to the cost. In this context, the much more affordable g‐CN, with its facile synthesis and intimate deposition techniques,^[^ ^92^ ^]^ emerges as a promising solution. It can be easily re‐coated onto 3C‐SiC PC for extended operational use, bringing down the overall cost of the electrode.

Textured g‐CN has been grown, printed and deposited on surfaces of various substrates with film thicknesses ∼50 nm to 50 µm (see ref. [92] and references therein). Since g‐CN has inherently large resistivity,^[^ ^85^ ^]^ its thickness must be small enough to enable efficient charge transfer, while sufficiently large to protect the the 3C‐SiC PC from photocorrosion. For the widely studied silicon photoelectrode, a 4–143 nm‐thick corrosion‐resistive TiO_2_ coating prevents corrosion and promotes carrier transport.^[^ ^151^ ^]^ Therefore, we assume a thickness of ∼50 nm of the g‐CN top layer. The thermal synthesis of g‐CN requires ∼550 °C temperature, while a SiC device permits an operation environment at up to ∼1000 °C,^[^ ^9^ ^]^ much higher than silicon. This makes the fabrication of g‐CN/3C‐SiC heterojunctions feasible.

A carbon buffer layer might be beneficial for reducing the junction resistance based on the study of graphene/3C‐SiC heterojunction.^[^ ^83^ ^]^ This may help offset the poor carrier transport of g‐CN. Indeed, carbon nitrides have been deposited on mesoporous carbon and graphene to increase the conductivity and electron transfer.^[^ ^152^, ^153^ ^]^ A g‐CN sheet has large structural voids, forming vertical channels along the stacking direction and making it difficult to achieve full protection with thin coating layer. A carbon buffer can reinforce the coverage of 3C‐SiC PC against photocorrosion. Increasing the sample quality of g‐CN is crucial for reducing deep traps and improving the charge carrier diffusion lengths.^[^ ^147^, ^154^ ^]^ We assume a high‐quality crystalline layer^[^ ^146^ ^]^ with few grains boundaries close to the 3C‐SiC region. The deposition of a porous layer in the exterior region of the g‐CN coating^[^ ^149^ ^]^ can add to the surface roughness, hold cocatalyst nanoparticles if necessary, and expose more reaction sites for water oxidation. Indeed, surface roughness and nanoporosity on 3C‐SiC by electrochemical etching has been implemented to enhance PEC efficiency.^[^ ^24^, ^25^ ^]^

## Appendix E Feasibility of NiO/3C‐SiC Heterojunction Photonic Crystal Photoanode

While very few OER catalysts (mostly noble metal‐based materials such as Ir or IrO_2_) can reliably operate in acidic environment,^[^ ^82^, ^155^ ^]^ some first‐row transition metal oxides, including NiO and cobalt(II, III) oxide (Co_3_O_4_), hold high activity and strong stability in basic environments.^[^ ^100^, ^101^, ^103^, ^156^, ^157^, ^158^, ^159^, ^160^ ^]^

Non‐stoichiometric NiO is an Earth‐abundant, p‐type (due to intrinsic, unintentional Ni^2+^ vacancies) semiconductor with rhombohedral or cubic structures.^[^ ^161^ ^]^ The band diagram of NiO is shown in Figure 1b. Within the bandgap of ∼3.7 eV, there are d‐d transitions at ∼1 eV above the VBM which may act as recombination centers.^[^ ^162^, ^163^ ^]^ The Fermi level is ∼0.4–1 eV^[^ ^108^, ^110^, ^162^ ^]^ above the VBM. The resistivity of NiO increases with its stoichiometry, which can vary from ∼10^−4^ to ≳ 10^4^ Ω cm^[^ ^106^, ^107^, ^164^ ^]^ and is tunable by oxygen supplies^[^ ^164^ ^]^ and extrinsic dopants^[^ ^165^ ^]^ during the sample preparation.

In addition to its broad range of applications in transparent diodes, gas sensors, solar cells and electrochromic devices, NiO_*x*_ has also been employed as an anti‐corrosion coating of unstable semiconductor photoanodes such as Si, GaN, CdTe, and InP^[^ ^99^, ^100^, ^101^, ^102^, ^103^ ^]^ due to its remarkable durability in basic solution.^[^ ^166^ ^]^ Stable OER performances for >300  and 1200 h at ∼10  and ∼30 mA cm^−2^, respectively, in alkaline media were demonstrated,^[^ ^100^, ^160^ ^]^ and ∼2200 days operation for 50 nm coating film could be expected according to the extrapolation of the Ni leaching rate.^[^ ^100^ ^]^ Apart from the slow dissolution of Ni, the degradation is primarily caused by the formation of a self‐passivation layer of higher nickel oxidation states,^[^ ^103^ ^]^ which can be reversed by an in situ periodic cyclic voltammetry, achieving 85% stability over 1000 h with ∼10 mA cm^−2^ photocurrent density.^[^ ^103^ ^]^

As for the activity, NiO and its in situ formed electrolyte‐permeable oxyhydroxide NiOOH are moderate OER catalysts.^[^ ^100^, ^156^ ^]^ However, incorporating Fe impurities can dramatically increase the activity of NiO to an order of magnitude higher than noble metal oxides.^[^ ^167^ ^]^ Indeed, the resulting Ni(Fe)O_*x*_H_*y*_ has demonstrated the highest OER activity among the first‐row transition metal (oxy)hydroxides in alkaline environment.^[^ ^156^ ^]^ Such Fe impurities can be unintentionally introduced to the NiO surface due to their abundance in alkaline electrolytes, unless specifically purified.^[^ ^100^, ^156^, ^168^ ^]^

The thickness of the protective coating should balance anti‐corrosion effectiveness with the possible carrier recombination loss introduced by the film. In this study, we select a thickness of 50–75 nm, based on experimental studies showing strong protection of ∼75 nm‐thick coating on unstable silicon^[^ ^101^, ^160^ ^]^ and numerical results on light‐trapping efficiency (see Figure 7).

## Appendix F Structure Model of Slanted Parabolic‐Pore PC

A “slanted parabolic pore” can be conceived and numerically implemented in different ways. This appendix provides the rigorous definition of the term as we use it. A normal parabolic pore [rotation axis (0, 0, 1)] with base radius *R*, axial depth *h* and apex *P*_A_(0, 0, *z*_apx_) is described by

(F1)
$$\begin{array}{c} z-z_{\text{apx}}=\frac{h}{R^{2}}\left(x^{2}+y^{2}\right) \end{array}$$

and depicted in **Figure** **F1**a. The slanted parabola pore is obtained by rotating such a normal parabola pore around a point *P*_0_(*x*_0_, 0, *z*_0_) by a slant angle θ in the *x* − *z* plane (see Figure F1b).

For numerical simulation, the normal parabolic pore is implemented by a stack of elementary cylinders, centering at *P*_1_(0, 0, *z*_1_) with orientations ${\hat{n}}_{1}=\left(0,0,1\right)$, radii $r=R\sqrt{(z_{1}-z_{\text{apx}})/h}$, and thickness along ${\hat{n}}_{1}$ equal to one FDTD mesh size. Similarly, the slanted parabolic pore is modeled by a stack of elementary cylinders centering at *P*_2_(*x*_2_, *y*_2_, *z*_2_) with orientations ${\hat{n}}_{2}=\left(-\sin\theta ,0,\cos\theta \right)$ and the same radii and thickness (along ${\hat{n}}_{2}$) as normal pores. Here *x*_2_ = *x*_0_(1 − cosθ) − (*z*_1_ − *z*_0_)sinθ, *y*_2_ = 0, and *z*_2_ = *z*_0_ + (*z*_1_ − *z*_0_)cosθ − *x*_0_sinθ. In this work, we consider a rotation point *P*_0_(−*R*, 0, *z*_0_) where *z*_0_ is the *z*‐coordinate of the base of the normal parabolic pore. The slanted parabolic‐pore PC is obtained by filling all regions exterior to a square lattice array of such pores, of lattice constant *a*, with SiC and interior regions with a thin coating layer, if any, and aqueous solution elsewhere.

The vertical depth *h*′ of the slanted (rotated) parabolic pore depicted in Figure F1 is obtained as follows. Let *P* = *P*(*x*′, *z*′) be a point in the *x*‐*z* plane in the co‐rotated frame of the parabola pivoting around *P*_0_(*x*_0_, 0, *z*_0_), and *P* = *P*(*x*, *z*) represent the same point in the original, non‐rotated frame. The coordinates transform according to *x*′ = *x*_0_ + (*x* − *x*_0_)cosθ + (*z* − *z*_0_)sinθ and *z*′ = *z*_0_ + (*z* − *z*_0_)cosθ − (*x* − *x*_0_)sinθ. For a 2D parabola in the *x*‐*z* plane in the co‐rotated frame *z*′ = *kx*′^2^, the equation of the parabola in the non‐rotated frame becomes

(F2)
$$\begin{array}{cc} z= & z_{0}-\left(x-x_{0}\right)\frac{\cos\theta }{\sin\theta }-x_{0}\frac{1}{\sin\theta }+\frac{1}{2k}\frac{\cos\theta }{\sin^{2}\theta }\\ & \pm \frac{1}{2\sqrt{2}k\sin^{2}\theta }\left[1+4kz_{0}+\left(1-4kz_{0}\right)\cos2\theta \right.\\ & {\left.-8k\left(x-x_{0}\right)\sin\theta -4kx_{0}\sin2\theta \right]}^{1/2} \end{array}$$

The minimum point of the rotated parabola, denoted by *P*_min_(*x*_min_, *z*_min_) in Figure F1, is determined by solving *dz*/*dx* = 0, where *z*(*x*) is given by the lower branch of Equation (F2). The vertical pore depth is then obtained by *h*′ = *h* − *z*_min_, yielding

(F3)
$$\begin{array}{cc} h^{\prime }= & h-z_{0}+z_{0}\cos\theta +x_{0}\sin\theta +\frac{1}{4k}\frac{\sin^{2}\theta }{\cos\theta } \end{array}$$

where *h* is the axial pore depth of the parabola and θ ∈ [0, π/2) is the slant angle. In this work *k* = *h*/*R* ^2^ and the rotation point locates at *P*_0_ = *P*_0_(−*R*, 0, *h*), i.e., *x*_0_ = −*R* and *z*_0_ = *h*. For small slant angle θ, the vertical pore depth is *h*′ ≈ *h*cosθ.

## Appendix G Convergence Analysis of the FDTD Simulation

To obtain a reliable FDTD simulation result, the mesh resolution and the simulation time must be suitably chosen to ensure convergence. In **Figure** **G1**a we examine the MAPD as a function of the lattice constant *a* by varying the mesh size of the FDTD simulation for g‐CN/3C‐SiC spbPore PC architecture. It is seen that the results obtained by 10 nm‐resolution is very close to that of 5 nm‐resolution (suggesting convergence), while resolutions >20 nm reveal substantial deviations. A mesh size with scaling property, namely, equal to *a*/70, is also compared in the same figure. Such resolution has been used to simulate TiO_2_ slanted conical‐pore PC.^[^ ^33^, ^34^ ^]^ The results from *a* = 0.25 µm to 1.5 µm match for mesh size 10  and 5 nm. Figure G1a shows the importance of choosing a fine enough mesh size in the simulation.

The exit criterion (time steps required for convergence) of our simulation is the decay of the *x*‐component of the electric field, *E*_*x*_, at the center of the PC unit cell to 1 × 10^−7^ of its peak value. In a few cases (the thicknesses of *d*_PC_ = 44, 46, 48, 50 µm in Figure 3) we relaxed the exit criterion to a few times of 10^−7^ due to the intense computational time and memory required for simulating thick PC structures with fine mesh resolution (10 nm) and weak material absorption. Nevertheless, an exit criterion of ∼10^−7^ *E*_*x*_ decay factor is more than enough to reach the convergence of the FDTD simulation and obtain a stable absorptivity spectrum. For example, in Figure G1b we plot a typical dependence of the implied MAPD with physical light propagation time in the simulation, and the corresponding *E*_*x*_ decay factor. It shows that the value of MAPD stabilizes when the *E*_*x*_ decay factor decreases to ∼10^−4^–10^−5^.
